# Deregulation of Lipid Homeostasis: A Fa(c)t in the Development of Metabolic Diseases

**DOI:** 10.3390/cells9122605

**Published:** 2020-12-04

**Authors:** Sabina Cisa-Wieczorek, María Isabel Hernández-Alvarez

**Affiliations:** 1 Laboratory of Hematology, Department of Hematology, Hospital de la Santa Creu i Sant Pau, Universitat Autònoma de Barcelona, IIB Sant Pau, 08041 Barcelona, Spain; scisa@santpau.cat; 2 Departament de Bioquímica i Biomedicina Molecular, Facultat de Biologia, Universitat de Barcelona, 08028 Barcelona, Spain; 3 Centro de Investigación Biomédica en Red de Diabetes y Enfermedades Metabólicas Asociadas (CIBERDEM), Instituto de Salud Carlos III, 28029 Madrid, Spain

**Keywords:** dietary lipids, phospholipids synthesis, metabolism, mitofusin-2

## Abstract

Lipids are important molecules for human health. The quantity and quality of fats consumed in the diet have important effects on the modulation of both the natural biosynthesis and degradation of lipids. There is an important number of lipid-failed associated metabolic diseases and an increasing number of studies suggesting that certain types of lipids might be beneficial to the treatment of many metabolic diseases. The aim of the present work is to expose an overview of *de novo* biosynthesis, storage, and degradation of lipids in mammalian cells, as well as, to review the published data describing the beneficial effects of these processes and the potential of some dietary lipids to improve metabolic diseases.

## 1. Introduction

Obesity has reached global epidemic proportions in both adults and children and is associated with significant morbidity and mortality. Obesity and other diseases such as diabetes, hypertension, and hyperlipidemia constitute a collection of issues, known as metabolic syndrome, that are known risk factors for coronary artery disease, the most common cause of heart failure [1,2]. Obesity refers to an abnormal or excessive accumulation of fat that impairs health. Lipids are important fats that perform different roles in the human body; however, an excess of fatty acids (FAs) can be harmful to cells and lipid homeostasis is crucial to prevent lipotoxicity. Fat-rich foods naturally have a high caloric density; therefore, they are a convenient source that yield high energy [3]. FAs are a major fuel for different types of cells. Lipids are essential for crucial biological functions by acting as energy stores, structural components of cell membranes, and important signaling molecules [4]. The synthesis and degradation of lipids are finely orchestrated by energy and metabolic balance. When the uptake of FAs exceeds a cell’s demand, excess FAs are esterified into neutral lipids and stored in specialized organelles called lipid droplets (LDs) [5]. LDs prevent lipotoxicity and regulate cellular processes such as membrane and lipid trafficking, endoplasmic reticulum (ER) stress, and protein storage and degradation [6].

The goal of this review was to assess the potential of lipids to function as biomarkers in metabolism. For this, we discussed how these lipids are synthetized *de novo* from precursors in mammalian cells and how they can be incorporated by the diet. We detailed the recycling of lipids by cellular processes like lipophagy. In addition, we included aspects of lipid metabolism that could be clinically targeted to control metabolism rather than being simply associated with defects in diseases. In all, we propose the lipid metabolism and, more specifically, the phospholipid metabolism as a direct and potent regulator of human health.

## 2. Lipids: Diet Incorporation, Biosynthesis and Storage

### 2.1. Triglycerides

**Figure 1:**
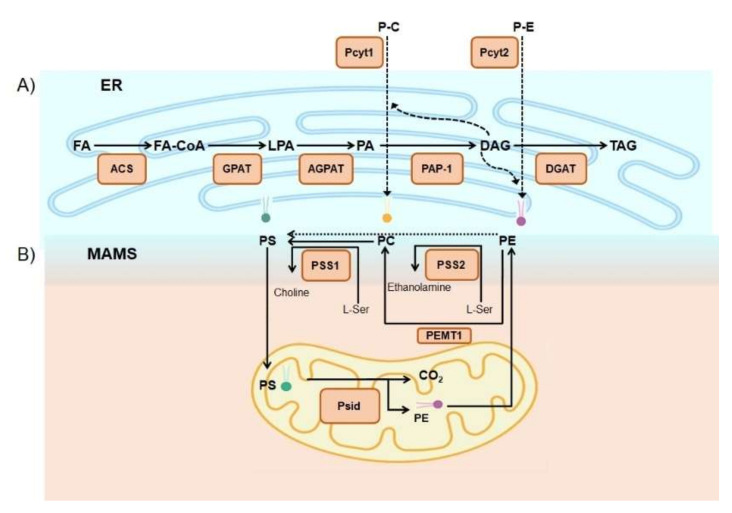
(**A**) Triglycerides (TAG) are synthetized by lipogenesis in the endoplasmic reticulum (ER) by esterification of three fatty acids with one molecule of glycerol. Lipids and enzymes (ER panel from left to right): FAs, fatty acids; FA-CoA, fatty-acyl-CoA; GPAT, glycerol-3-phosphate acyltransferase; LPA, lysophosphatidic acid; AGPAT, 1-acylglycerol-3-phosphate acyltransferase; PA, phosphatidate; PAP-1, phosphatidic acid phosphate; DAG, diacylglycerol; DGAT diacylglycerol acyltransferase. (**B**) Phospholipid synthesis *de novo* in mammalian cells takes place between the endoplasmic reticulum by the cytidine diphosphate (CDP)–Kennedy pathway and in mitochondria-associated membranes (MAMs), as discussed in the text. Phospholipids and enzymes from upper to bottom panel: P-C, phosphocholine; P-E, phosphoethanolamine; Pcyt1, choline-phosphate cytidylyltransferase; PCyt2, ethanolamine-phosphate cytidylyltransferase; PS, phosphatidylserine; PC, phosphatidylcholine; PE, phosphatidylethanolamine; PSS1, PS synthase-1; PSS2, PS synthase-2; PEMT1, phosphatidylethanolamine N(PEN)-methyl transferase; Psid, mitochondrial phosphatidylserine decarboxylase.

There are different forms of lipids: triacylglycerol (or triglycerides), phospholipids, sterols, and eicosanoids (biologically active lipid mediators). The triglycerides are the most abundant lipids in dietary fat intake [7]. Triglycerides (TAG) are commonly found in fried foods, vegetable oil, butter, whole milk, cheese, cream cheese, and some meats [8]. In addition, in mammalian cells, TAG can be produced *de novo* in mitochondria and the ER by lipogenesis. TAG formation in the pathway of lipogenesis involves the esterification of three fatty acids with one molecule of glycerol and is a way to store energy. The first step in this pathway is the action of the fatty acyl-coenzyme A (CoA)synthetase (ACS) that catalyzes the ATP-dependent formation of a thioester bond between a fatty acid and Coenzyme A. Next, the lysophosphatidic acid (LPA) is generated when the glycerol-3-phosphate acyltransferase (GPAT) esterifies one fatty acid-CoA to the glycerol 3-phosphate backbone. From there, the phosphatidate (PA) is synthesized *de novo* from the acylation of LPA in a reaction catalyzed by acyl-CoA:1-acylglycerol-sn-3-phosphate acyltransferase (AGPAT). At this point, diacylglycerol is produced from PA in a reaction catalyzed by phosphatidic acid phosphatase (PAP). It is important to highlight that diacylglycerol (DAG) settles at the branch point between phospholipids like phosphatidylcholine and phosphatidylethanolamine, and TAG synthesis (Figure 1A). The regulation of DAG flux is strongly influenced by cytidine triphosphate (CTP):phosphocholine cytidylyltransferase activity and the requirement for phosphatidylcholine (PC) synthesis [9]. Finally, the formation of TAG can be catalyzed by several different activities, including diacylglycerol: acyl-CoA acyltransferase (DGAT) [10,11] (Figure 1A).

### 2.2. Phospholipids

**Figure 2:**
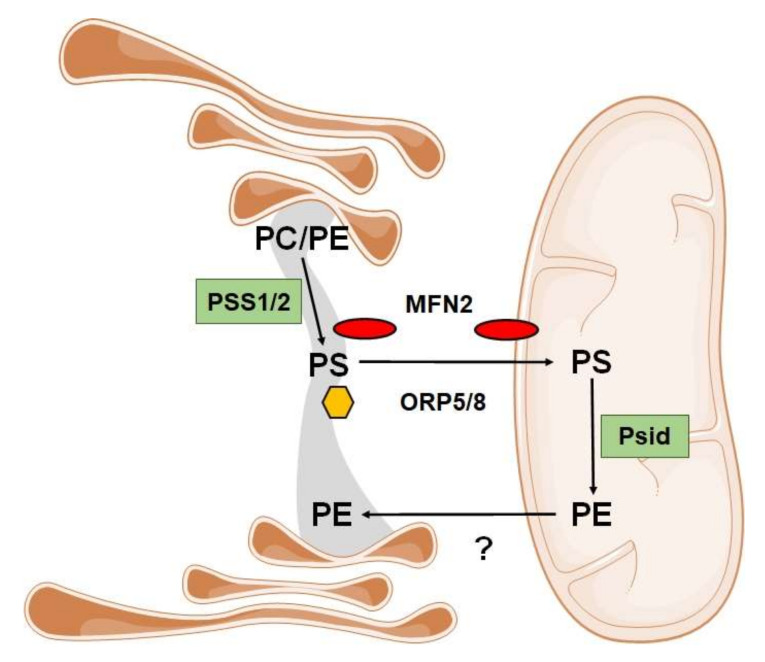
Phosphatidylserine synthetized into the endoplasmic reticulum can be transferred into mitochondria through MAMs (mitochondria-associated membranes). PC, phosphatidylcholine; PE, phosphatidylethanolamine; PS, phosphatidylserine; MFN2, mitofusin 2; ORP5/8, oxysterol-binding protein-related protein 5/8.

Phospholipids (PL) are the second main type of dietary lipids. Dietary PLs have beneficial effects on a range of human diseases and conditions, such as coronary heart disease, cancer, or inflammation [12]. PLs are present in almost all types of food, being particularly abundant in egg yolk, soybean, and in dairy products, such as milk [12]. In this regard, the major phospholipids present in milk are phosphatidylethanolamine (35.7%), phosphatidylcholine (26%) and sphingomyelin (23%), whereas phosphatidylserine (7.2%) and phosphatidylinositol (6.3%) are present in smaller amounts [13]. PLs can also be synthesized *de novo* in almost all tissues, including the brain, although PL synthesis and metabolism were found to take place predominantly in hepatic tissue [14]. PLs are the major component of cell membranes. The most abundant phospholipid in mammalian cells is phosphatidylcholine (PC), followed by other phospholipids, including phosphatidylethanolamine (PE) and phosphatidylserine (PS), which are biosynthesized in cells as well. The synthesis of these three mammalian phospholipids mostly takes place in the endoplasmic reticulum, with PA and DAG as precursors (Figure 1). PS is synthesized in the ER by the exchange of serine for the choline or ethanolamine head-groups of PC or PE by PS synthases. The PS synthases, which are enriched in a specific domain of the endoplasmic reticulum (ER) called mitochondria-associated membranes (MAMs), catalyze parallel base-exchange reactions in which serine replaces the choline or ethanolamine head group of PC (PS synthase-1; PSS1) or PE (PS synthase-2; PSS2), respectively. Newly made PS is transferred into the mitochondria through MAMs, where it is decarboxylated to PE via PS decarboxylase in the mitochondrial inner membrane [15] (Figure 1B). Recently, it has been proposed by our group that PS transfer at MAM interfaces is facilitated by mitofusin 2 and/or by oxysterol-binding protein-related protein 5 (ORP5) and ORP8, which are also localized in MAMs according to Galmes and collaborators [16,17] (Figure 2).

When PS is localized at the mitochondria, it can be decarboxylated by a specific decarboxylase (mitochondrial phosphatidylserine decarboxylase, Psid) to form mitochondrial PE [18]. In mammalian cells, the CDP–ethanolamine pathway can also produce PE. This pathway requires the dietary intake of ethanolamine, which is converted to PE by three enzymatic steps ending at the ER. Because of the different origins of PE biosynthesis, and because the PE made in the ER is not efficiently imported into mitochondria, the pools of PE made in the ER and mitochondria are functionally distinct. Indeed, PE in the mitochondrial membranes is primarily made *in situ* in mitochondria from PS decarboxylation, rather than being imported from the ER [19] (Figure 1B). The synthesis of PE in mitochondria is so important that the elimination of PS decarboxylase activity in mice is lethal, despite the proper function of the CDP–ethanolamine pathway [20]. Moreover, ablation of Mfn2 in the liver causes a deficient transference of PS from the ER to mitochondria with a following reduction of mitochondrial PE synthesis. This specific defect causes TAG accumulation, ER stress, mitochondrial dysfunction, the non-alcoholic steatohepatitis (NASH)-like phenotype, and liver cancer with age [16].

The major site of biosynthesis of phosphatidylcholine is the ER, where PC can be produced via two different pathways in mammals. PC is primarily synthesized via the cytidine diphosphate (CDP)–choline pathway in a similar manner to CDP–ethanolamine. In the second route, PC synthesis depends on the conversion of PE (which comes from the mitochondria) to PC by three sequential methylation reactions, all of which are catalyzed by phosphatidylethanolamine N(PEN) -methyl transferase (PEMT1) in the ER membranes. In this regard, the protein or proteins involved in the transference of PE from mitochondria to the ER remain unknown (Figure 1B). Moreover, PEMT is quantitatively more important in the liver because PEMT activity (per mg protein) in other mammalian tissues is usually lower than 1% of its hepatic activity [15,21].

### 2.3. Sterols

Sterols are the least common type of lipid. Cholesterol is perhaps the best-known sterol. Sterols are synthetized in the ER and transported to other cellular locations, especially to the plasma membrane (PM), where cholesterol represents almost 40% of lipids. Cholesterol biosynthesis is divided into two major pathways: pre-squalene cholesterol synthesis and post-squalene cholesterol synthesis. Pre-squalene cholesterol synthesis contributes to both sterol and isoprenoid synthesis, whereas post-squalene cholesterol synthesis is a committed pathway to sterol and vitamin D synthesis [22]. The main food sources containing cholesterol are egg yolks, shrimp, beef, pork, and poultry, as well as cheese and butter [23].

### 2.4. Eicosanoids

Eicosanoids are formed from FAs found within cell membranes. First, these FAs are released from the membrane and subsequently converted into intermediate molecules, which are further metabolized to bioactive eicosanoids through different pathways. There are several subfamilies of eicosanoids; the best known members include prostaglandins (PGs), leukotrienes (LTs), and thromboxanes (TXAs), which are all derived from the same progenitor molecule, the arachidonic acid (AA) [24]. Eicosanoids are synthesized *de novo* when cells are activated by various cellular stimuli and they are never stored. Eicosanoid biosynthesis initiates with the desaturation of linoleic acid (LA) to form dihomo-γ-linolenic acid (DGLA) (20: 3) by the action of delta 6 desaturase and via the formation of γ-linolenic acid (GLA). AA is then produced by a delta 5 desaturase from the GLA [25]. Dietary GLA gets elongated to DGLA by the action of elongases however, DGLA can also be degraded by cyclooxygenases [26]. Unlike dietary GLA, AA directly obtained via the diet is stored within the cell membrane by the action of lysophospholipid acyltransferase and arachidonyl CoA. The enzyme phospholipase A2 (PLA2) then releases AA from the storage sites of the cell membrane and activates the action of the eicosanoid biosynthesis enzymes. This process is regulated by various types of phospholipase A2 (PLA2) [24]. AA is particularly obtained from meat products including chicken, beef, pork, and fish [27].

### 2.5. Lipid Droplets

**Figure 3:**
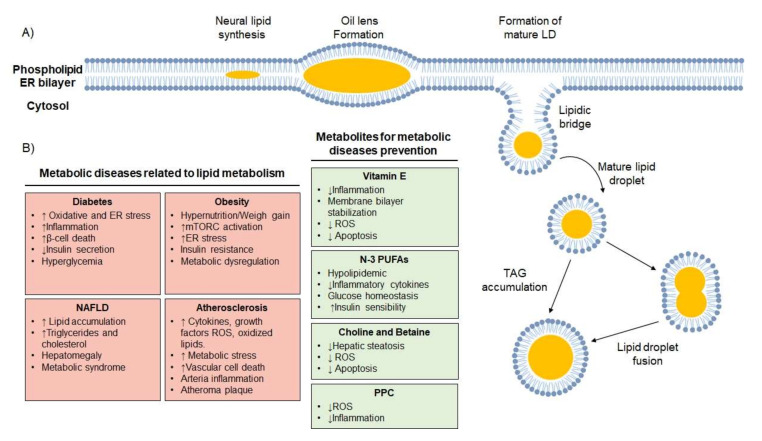
(**A**) Lipid droplet synthesis. Neutral lipids are synthesized into the endoplasmic reticulum (ER) and accumulate between leaflets to form lipid lenses. Lipid accumulation deforms the ER until the lipid droplet (LD) buds from the ER to cytosol. LDs can increase its size by fusion with other LDs or TAG local accumulation. (**B**) Relevant metabolic diseases associated with increased lipid metabolism and lipids with a beneficial effect on metabolic diseases.

Lipids are stored in specialized organelles known as lipid droplets (LDs). LDs are formed by a core of TAG and/or a sterol surrounded by a phospholipid monolayer with the hydrophobic phospholipid tails oriented towards the neutral core, providing an amphipathic interface with the cytosol and controlling the selective binding of proteins to the LDs [28]. Various proteins associated with the phospholipid monolayer influence LD metabolism and signaling. They are known generically as perilipins or lipid droplet-associated proteins (PLINs) [29]. LDs can be formed *de novo* or derived from existing LDs by fission. LD production is regulated in part by substrate availability and flux through the reactions. The model for *de novo* LD formation consists of three stages: neutral lipid synthesis, lens, and drop formation. In the first stage, the majority of the enzymes in charge of neutral lipid synthesis are localized at the ER to produce neutral lipids [30]. In the second stage, neutral lipids accumulate between the leaflets of the ER to form lipid lenses at pre-existing sites marked by a LD-targeted protein. This suggests that lenses form at the sites of previous LDs. Finally, emerging lipid lenses reach a certain size, causing the ER to deform. This allows the nascent LD to recruit specific proteins and to finally bud off from the ER via a dewetting mechanism to form a new structure [31] (Figure 3A).

LDs vary greatly in size, ranging from 20–40 nm to 100 mm in white adipocytes occupying the entire cytoplasm. The size of LDs not only depends on the cell type; under conditions of lipid overload, cells can respond by increasing the number and/or volume of the pre-existing LDs. Whereas giant LDs provide the most efficient form of fat storage, small LDs facilitate lipolysis by providing more surface area for lipases [32].

LDs can increase in size by increased TAG accumulation or by fusion with other LDs. When TAG synthesis increases, LDs are often found in close proximity to the ER and have been found connected to it through ER–LD membrane bridges through surface coatomer protein (COP)-I [33]. COPI proteins act at LDs surfaces by removing phospholipids and, as a result, the hydrophobic content of LDs becomes exposed, increasing the LDs surface tension to the aqueous environment and promoting the fusion of LDs with other membranes [34]. After ER–LD bridge establishment, specific isoforms of TAG synthesis enzymes use these connections to translocate from the ER to LDs [30]. Under conditions of LD expansion, CTP:phosphocholine cytidylyltransferase is translocated from the cytosol to LD surfaces and PC synthesis increases.

Pre-existing LDs can also increase their size by fusion with other LDs, a process mediated by cell death-inducing DFF45 like effector (CIDE) proteins, which have been found to be enriched and clustered at LD contact sites. CIDE proteins generate channels that allow lipid exchange between contacting LDs. Lipids are transferred from small to large LDs, due to higher internal pressure within small LDs [35].

Several proteins that interact with CIDE proteins have been identified. An example is PLIN1, a LD-associated protein that originally was found to protect LDs from lipase digestion. PLIN1 interacts with the *N*-terminal domain of CIDEc, a member of the CIDE family. PLIN1–CIDEc interaction promotes LD fusion and unilocular LD formation in adipocytes. PLIN1 stabilizes the CIDEc fusion complex by maintaining the active conformation of CIDEc, resulting in increased LD fusion efficiency [36].

In conclusion, TAG accumulation and/or fusion facilitated by CIDE-interacting proteins promote LD growth, whereas TAG degradation results in a reduction in LD size. Regulation of LD size and, consequently, storage capacity are important to maintain normal biological function, especially under starvation and stress conditions.

## 3. Dietary Lipid Metabolism in Metabolic Diseases

### 3.1. TAG Associated with Lipid Disorders

Disorders of lipid metabolism can produce a serious metabolic insufficiency, resulting in severe early-onset multisystemic diseases. For example, disorders affecting the utilization of stored TAG due to a defect of the triglyceride lipase are called neutral lipid storage diseases (NLSDs) [37]. NLSDs are rare autosomal recessive disorders characterized by excessive, non-lysosomal accumulation of neutral lipids in multiple tissues. Genetically, there are two different forms associated with defects of NLSDs: one is Chanarin–Dorfman syndrome (ABHD5 gene), which manifests as an ichthyosiform non-bullous erythroderma, which is a condition that mainly affects the skin, causing a tight, clear sheath covering the skin called a collodion membrane [37,38]. The second is a neutral lipid storage disease without ichthyosis associated with the PNPLA2 gene. Structural defects in the PNPLA2 gene mainly lead to myopathic symptoms that can present as distal muscle weakness [37].

Regarding dietary lipids, numerous studies have indeed provided evidence to support the pro-inflammatory role of saturated fats compared with unsaturated fats [39], and have demonstrated the mechanisms of how saturated fats are linked to metabolic diseases. Although intake of unsaturated fats is recommended over saturated ones, overconsumption of unsaturated fats can also negatively affect health [40]. In this regard, patients with metabolic risk have demonstrated a significant reduction in TAG plasma levels by intake or supplementation with n-3 poly-unsaturated fatty acids (PUFAs) [41]. This hypolipidemic effect of n-3 PUFAs is due to decreased lipogenesis, resulting in lower TAG release into circulation, in the form of very-low-density lipoproteins (VLDLs) secreted by the liver [42]. In addition, dietary supplementation with n-3 PUFAs in healthy subjects is associated with lower production of pro-inflammatory cytokines IL-1, IL-6, TNF-α, and TNF-β in response to an inflammatory stimulus [43]. Furthermore, n-3 PUFA supplementation prevents alterations in glucose homeostasis and development of Type 2 diabetes, improving insulin sensitivity [44,45] (Figure 3B).

### 3.2. Cholesterol Associated with Lipid Disorders

The intake of dietary cholesterol is usually associated with an enhanced intake of saturated fatty acids, that increases LDL (low-density lipoprotein) cholesterol levels and as a consequence, the risk of cardiovascular disease [46]. However, cholesterol has several biological functions, like the synthesis of lipid rafts, which are needed for protein sorting, cellular signaling, and apoptosis [47]. In addition, there is insufficient evidence to determine whether lowering dietary cholesterol reduces LDL cholesterol [48]. Several randomized controlled trials indicated that egg consumption increased HDL (high-density lipoprotein) cholesterol and decreased the risk factors associated with metabolic syndrome [49]. Along the same lines, Fuller and colleagues reported that consumption of a high-egg diet in pre-diabetes and patients with Type 2 diabetes who had energy-restricted diets had no adverse effect on blood glucose or glycated hemoglobin [50].

Epidemiological studies have shown that populations consuming large amounts of saturated atherogenic FAs have elevated levels of LDL cholesterol [51,52]. However, the highest atherogenic potential comes from the *trans*-fatty acids (TFA), which are obtained from partial hydrogenation of plant oils. TFA are characterized by the presence of one or more double bonds in *trans* configuration instead of the usual *cis* configuration and they lead to an increase in total and LDL cholesterol, and lower levels of HDL cholesterol [53]. Due to these effects, TFA are more atherogenic compared with saturated FAs. Furthermore, HDL levels in patients with metabolic syndrome encounter reductions in both the large and small HDL subclasses (HDL2 and HDL3). As the number of metabolic syndrome components increases, the HDL phenotype includes a greater percentage of small HDL3s and fewer large HDL2s, thus resulting in a lower HDL2/HDL3 ratio. Interestingly, an increased HDL2/HDL3 ratio has been observed after fish oil supplementation [54]. Moreover, this was related to a decrease in levels of plasma TAG: HDL particles become larger, retain more cholesterol, and are less susceptible to catabolism by the hepatic and renal clearance pathway [55]. Dietary intake influences changes in FA profiles and endogenous metabolism in different tissues. FAs play a role in the modulation of membrane fluidity, interact with intracellular signaling pathways, and act as substrates for producing signaling molecules [56].

### 3.3. Eicosanoids Associated with Lipid Disorders

Eicosanoids regulate a wide selection of physiological processes, ranging from inflammatory processes such as asthma and allergies to immune regulation and cancer. Altered lipid homeostasis in chronic diseases is associated with chronic inflammation in many organs and tissues such as the liver, adipose tissue, muscle, heart, and gastrointestinal tract [57]. Chronic inflammation is a feature of obesity and metabolic syndrome and results in metabolic tissue stress. In overnutrition, adipocytes accumulate more fat, resulting in cellular hypertrophy [58] involving ER-mitochondrial and oxidative stress. Persistent inflammation can lead to adipocyte apoptosis and the release of chemotactic mediators, resulting in infiltration of macrophages and leukocytes [59]. Subtypes of eicosanoids such as PGs, TXAs, and LTs are related to the chronic inflammation observed in metabolic diseases, including obesity, diabetes, non-alcoholic fatty liver disease (NAFLD)/NASH, or atherosclerosis [60]. The accumulation of FAs in obese patients increases the production of pro-inflammatory eicosanoids such as PGE2 and hydroxy-eicosatetraenoic acids (HETEs), LTs, and DAGs, which are implicated in insulin resistance [61]. An altered AA to eicosatetraenoic acid ratio has been found in the plasma of patients suffering from metabolic syndrome [62]. Diabetic and obese patients are prone to infections, with AA and its derivatives being the link between nutrient metabolism, inflammation, and immunity [63]. It has been shown that the increased production of pro-inflammatory molecules impairs leukocyte function, increases the survival of polymorphonuclear neutrophils, and decreases the activity of macrophages at inflamed sites in obese and diabetic patients [61]. In addition, low FA concentrations increase the proliferation of T-cells, whereas higher concentrations induce T-cell apoptosis in a dose-dependent manner, given that an overdose of FAs is toxic to T-cells. Furthermore, the degree of saturation and the length of FAs affect T-cell viability. While short FAs fail to be toxic even in high concentrations, longer and more saturated FAs are already toxic in low concentrations [64]. It is possible that FAs diffuse through the membrane of T-cells by a passive process or through an active process that involves fatty acid transport protein (FATP) and fatty acid binding protein (FABP) receptors [65]. Treatment of T-cells with FAs increases neutral lipid accumulation, which induces apoptosis by mitochondrial depolarization, cytochrome c and PS externalization, DNA fragmentation, chromatin condensation, and caspase activation [66].

### 3.4. Phospholipids Associated with Lipid Disorders

PLs are crucial for building the protective barrier of membranes. In the liver, deficiencies in vitamins cause potent defects in phospholipid metabolism. For example, vitamin E is converted to a tocopheroxyl radical, which reacts with other peroxyl radicals to form non-radical products. Furthermore, vitamin E is thought to stabilize membrane bilayers due to colocalization with PC [67] and cholesterol (leading to an association with lipid rafts) [68]. This ability of vitamin E is especially important in NASH [69]. In fact, supplementation with vitamin E in an animal model of NASH significantly reduced NF-κB signaling and the expression of markers for cell apoptosis such as Bax and Bcl-2 [70]. Additionally, deficiencies in vitamin A cause a significant decrease in total phospholipid content in liver mitochondria from rats [71]. This suggest that membrane fluidity can lead to mitochondrial dysfunction due to a decrease in cardiolipin content, a phospholipid found exclusively in mitochondrial membranes. Loss of mitochondrial cardiolipin causes dysfunctional hepatocytes with decreased mitochondrial membrane potential, which is associated with mitochondrial oxidative stress, pathogenesis, and progression of different liver diseases. In addition, the electron transport chain enzymes, located at the inner mitochondrial membrane, bind PC and PE, which is required for reaching the optimum activity of Complexes I and III [72].

Many of the non-alcoholic fatty liver disease (NALFD) patients have unhealthy dietary habits characterized by overconsumption of fructose and soft drinks, lower consumption of fiber, overconsumption of meat, saturated fat and cholesterol; lower consumption of fish or omega-3 fatty acids or n-3 PUFAs; and low consumption of some vitamins [73]. Since PC is the major phospholipid within the coat of LDs and lipoproteins, there is a biological need for synthesis of PC when an excessive food intake stimulates lipogenesis for energy storage. PC is an essential structural component of VLDLs and is required for the secretion and export of TAG from the liver. Therefore, choline and subsequent PC deficiency may result in hepatic accumulation of lipids [74,75]. Indeed, studies have confirmed a decreased hepatic PC to PE ratio in NASH patients, as well as marked changes in FA composition in the livers of NAFLD patients. [75,76]. In addition, there are reports pointing out that dietary choline deficiency has multiple implications for human health, including birth defects, neurological dysfunction, and the development of fatty liver [77]. In animal studies, choline and betaine supplementation has been shown to reduce the hepatic steatosis associated with NAFLD, as well as the oxidative stress and apoptosis associated with liver damage featured in NASH [77]. Furthermore, it has been shown that after supplementation with polyenylphosphatidylcholine (PPC), the PL provided may be directly incorporated into the membrane of liver cells, regulating oxidative balance, inhibiting inflammatory factors, and suppressing the NF-κB signaling pathway [78] (Figure 3B).

Phospholipids also play important roles in other tissues besides the liver. For example, in muscle, a defect in the choline kinase (the first enzyme in the cytosolic biosynthetic pathway of PC) causes congenital muscular dystrophy with a peculiar mitochondrial abnormality. Mitochondria are markedly enlarged at the periphery of muscle fibers and are absent from the center [79]. Another example is muscle primary carnitine deficiency, which results from a defect of the carnitine transporter. Affected patients excrete filtered carnitine in the urine and suffer from late-onset and permanent muscle weakness [80]. Moreover, secondary forms of carnitine deficiency are also observed in several other muscle disorders, including acyl-CoA dehydrogenase deficiencies [81].

In the bone marrow, PLs are essential as FA pools because changes in the membrane phospholipid fatty acid composition may affect bone cell signaling and, potentially, bone mineralization [82]. It is known that an abundance of the essential omega-3- fatty acid member docosahexaenoic acid (DHA) within the brain’s phospholipid pool also serves to generate a protective eicosanoid: neuroprotectin D1 (NPD1), which has potent anti-inflammatory and neuroprotective bioactivity. NPD1 reduces Ab42 peptide release from aging human brain cells and is severely depleted in Alzheimer’s disease [83]. Long-term intake of a DHA-rich diet is also essential for maintaining and improving the ability of learning and memory in elderly humans [84]. Furthermore, low tissue levels of n-3 PUFAs in phospholipids, particularly DHA, are implicated in postpartum depression. Brain regions of parous female rats fed a diet containing inadequate n-3 PUFAs exhibited a regionally specific decrease in phospholipid DHA content that was not caused by either reproductive status or deficient diet alone, indicating that this effect was due to an interaction between the diet and the physiological status. Thus, specific neuronal systems may be differentially affected by the depletion of brain DHA in the postpartum organism [85]. The best sources of ω-3 DHA in the diet are seafood, algae, and especially cold-water fish and fish oil [86].

In blood and biofluids, PLs form structures in which lipids are enclosed and transported all over the bloodstream. Metabolically, phospholipids are broken down into lysophospholipids due to the hydrolysis of one acyl. This reaction is catalyzed in the small intestine by the pancreatic phospholipase A2 for PC, PE, PS, and PI and by the alkaline sphingomyelinase for sphingomyelin [87]. These lysophospholipids are absorbed by enterocytes in the gastrointestinal tract and then re-esterified into phospholipids in the chylomicrons, where they are released by exocytosis into blood circulation via lymphatic vessels [88]. Furthermore, different pathologies, such as pancreatitis, cancer, diabetes, and coronary heart disease, among others, can be associated with changes in plasma or serum fatty acid phospholipids. Serum oleic acid has been directly associated with olive oil, linoleic acid with sunflower oil, pentadecanoic acid with dairy products, n-3 long chain-PUFAs with fatty fish, and *trans*-monounsaturated fatty acids with manufactured foods [89,90]. Positive associations with diabetes were observed for stearic acid and total saturated fatty acids in plasma phospholipids, whereas an inverse association was reported for linoleic acid [91]. The importance of dietary and *de novo* biosynthetic phospholipids are beyond their own proper function as single molecules but also as part of organelles that control lipid overload and storage like the autophagosomes.

## 4. Potential Role of Autophagy in the Modulation of Lipid Metabolism

### 4.1. Phospholipid Dependence of Autophagosome Formation

Autophagy is a major degradative mechanism by which a cell degrades intracellular components. Initially, autophagy was regarded as a survival response to stress that allows cells to survive during stress conditions by regulating energy homeostasis and protein or organelle quality. In addition, recent studies have revealed its significance in cellular and organismal homeostasis, development, and immunity [92]. It is important for balancing sources of energy in response to nutrient stress and clearing damaged organelles, removing misfolded or aggregated proteins. Autophagy begins with formation of an isolation membrane (phagophore) that expands to engulf the cellular cargo, such as protein aggregates, sequestering the cargo in a double membrane forming the autophagosome. The autophagosome fuses with the lysosome, which provides lysosomal proteases, lipases, glycosidases, and nucleases that degrade the autophagosome content [93]. Once macromolecules have been degraded, monomeric units are exported to the cytosol for reuse for building macromolecules and for metabolism. Various intracellular sources including the endoplasmic reticulum exit sites (ERES), mitochondria, ER–mitochondria contact sites, Golgi apparatus, ER–Golgi intermediate compartment (ERGIC), the PM, LDs, and endosomes have been proposed to supply lipids to the autophagosome membrane. The ER is probably the major donor and it is possible that different membrane sources are utilized, dependent on the cell type, growth and physiological conditions, and the intended cargo [93].

Early autophagic structures have been found associated with ERES sites, regions of the ER where COPII transport vesicles are generated. The presence of phosphatidyl-inositol-3-phosphate (PI3P) and the ER-associated FYVE domain-containing protein (DFCP1) in these regions suggests that the omegasome isolation membrane, autophagosomes (AP), and phagophores probably derive from the ER [94]. Moreover, it has been shown that the autophagosome formation marker ATG5 localizes at ER–mitochondria contact sites and that PI3P accumulates in the MAMs, establishing a link between ER–mitochondria contact sites and autophagosome formation [95,96]. Importantly, when mitochondria contact sites are disrupted in Mitofusin 2 knock-out (KO) cells, autophagosome formation is drastically suppressed [97]. ATG5 has been found in mitochondria puncta, accompanied by the late autophagosomal marker, the autophagy-related protein microtubule-associated protein light chain 3 (LC3), suggesting that mitochondria play a central role in autophagy induction [97]. Several studies suggest that there is a connection linking mitochondrial outer membrane lipids, proteins, and AP formation. In this regard, it has been suggested that the outer mitochondrial membrane (OMM) supplies a flow of lipids and membrane proteins for AP formation [93]. In the same line, the transmembrane protein ATG9 is found at the Golgi apparatus and in endosomes, suggesting they might be involved in providing membrane components during the early stages of autophagosome formation [98]. Recently, ER–plasma membrane contact sites (ER-PMcs) have been found to play a role in Ca^2+^ signaling and lipid homeostasis. Nascimbeni and collaborators showed that ER-PMcs are mobilized for autophagosome biogenesis by direct implication of the tethering function of extended synaptotagmin (E-Syts) proteins, which are essential for autophagy-associated PI3P and, in consequence, for AP biogenesis [99]. Finally, ATG9-containing cytoplasmic vesicles [100], ER–lipid droplet (LD) contact sites [101], and COPII vesicles [102] are also considered sources of membranes for AP formation.

### 4.2. Metabolic Diseases Related to Defective Autophagy

Defective autophagy has been found in a large number of human diseases such as diabetes, obesity, liver disease, and atherosclerosis. Glycemia is regulated by the pancreatic islets of Langerhans, where β-cells secrete the hypoglycemic hormone insulin when glycemia increases, leading to glucose uptake by the liver via glycogenesis. Impaired autophagy induces oxidative, ER, and inflammatory stress and results in dysfunction and enhanced death of β-cells [103]. Patients with Type 2 diabetes mellitus (T2D) show autophagic vacuole accumulation and an increased number of autophagosomes. In addition, it has been described that rodent β-cells in insulin-resistant and diabetic models show increased numbers of autophagosomes [104]. In obesity, hypernutrition inhibits autophagy as a consequence of 5′ adenosine monophosphate-activated protein kinase (AMPK) inhibition and ultimately causes chronic mTORC1 activation [105]. In genetic and dietary mouse models, there is an autophagic downregulation; particularly, low levels of Atg7 expression have been observed in liver. Suppression of Atg7, *in vivo* and *in vitro*, results in insulin resistance and elevated ER stress, linking obesity and T2D [106]. In addition, in mouse models with a hepatocyte-specific deletion of Atg7, hepatomegaly and increased levels of liver triglycerides and cholesterol were observed [104].

Furthermore, autophagy stimuli such as inflammatory cytokines, reactive oxygen species (ROS), oxidized lipid species, growth factors, and metabolic stress are also related to atherosclerosis development. Atherosclerosis is a chronic arterial inflammatory disease characterized by the formation of lipid-containing plaques in the vessel wall of large and medium-sized arteries, ultimately leading to a complex plaque that impedes blood flow. In this regard, analysis of plaques from atherosclerosis patients show elevated LC3-II levels [107], and vascular smooth muscle cells isolated from carotid plaques show increased mitophagy [108]. However, in studies comparing symptomatic and asymptomatic patients, LC3-II levels decrease fivefold in symptomatic patients [109]. Supporting this concept, ApoE-null mice have increased levels of autophagic markers in early stages, whereas the progression of atherosclerotic plaque leads to autophagy deficiency [110]. It seems that autophagy is regulated depending on the disease stage. It is possible, that it has a beneficial effect by protecting plaque cells against oxidative stress, degrading damaged intracellular material, and promoting cell survival. In contrast, excessive autophagy stimulation might cause autophagic cell death, leading to reduced synthesis of collagen, thinning the fibrous cap, plaque destabilization, lesioned thrombosis, and acute clinical events [111].

## 5. Regulation of Lipid Stores and Metabolism by Lipophagy

Lipophagy is the specific autophagy process that degrades LDs and it has emerged as a significant component of lipid metabolism with important implications for human health. Impaired lipophagy has been observed in many diseases that produce an increase in cellular LDs. The objective of LD degradation is to provide a source for ATP, building materials for membrane synthesis or repair, precursors of hormones, and secondary messengers for intracellular signaling pathways [112]. On the other hand, LDs provide precursors for energy conversion, PL biosynthesis, and signaling molecules by lipolysis or selective turnover by autophagy [113]. First, the breakdown of LDs was attributed to lipolysis, a pathway mediated via lipid droplet lipases such as adipose triglyceride lipase (ATGL). However, new findings have demonstrated a distinct autophagic pathway to specifically degrade lipids, called lipophagy.

In lipophagy of LDs, TAGs and cholesterol are taken up by autophagosomes and delivered to lysosomes for degradation by acidic hydrolases in order to fuel cellular rates of mitochondrial β-oxidation. Lipophagy therefore functions to regulate intracellular lipid stores, cellular levels of free lipids such as FA, and energy homeostasis. Smaller lipid droplets may be engulfed completely by autophagosomes, whereas larger lipid droplets seem to be broken down into smaller droplets. In larger LDs, lipolysis and lipophagy are sequential pathways, whereby lipolysis reduces the size of large LDs to allow autophagic engulfment of small LDs for lipophagy [114].

Lipophagy was identified first in hepatocytes, cells prone to an excessive lipid accumulation and large intracellular lipid stores. Pharmacological inhibition of autophagy with 3-methyladenine in hepatocyte cell cultures increases TAGs and LDs, while mitochondrial β-oxidation is impaired. This is caused by decreased free FA generation, which is needed to sustain cellular rates of mitochondrial β-oxidation and levels of ATP [113]. *In vivo*, these results were confirmed in mice with a hepatocyte-specific ATG7 KO. ATG7 KO mice have increased hepatic TAG and cholesterol levels, due to impairments of the lipophagic pathway. These findings changed our view of lipid metabolism and provided an alternative mechanism of LD degradation, where autophagy-mediated lysosomal degradation participates in the cellular mobilization and metabolism of intracellular lipids from LDs. The ability of the cell to alter the amount of lipids targeted for autophagic degradation, depending on nutritional status, demonstrates that this process is selective. Components of the autophagic machinery have an alternative role in lipid metabolism. For instance, LC3, an essential protein for macroautophagy, is involved in LD formation. Another example is ATG7, which is required for LD formation upon fasting. Studies with isolated LDs display enrichment of cytosolic LC3-I and autophagosome-bound LC3-II, which suggests that the conversion of LC3-I into LC3-II occurs at the surface of LDs. Given the enormous size of LDs, it is possible that unconjugated LC3-I is localized to LDs, where it is converted to LC3-II by PE addition, thereby generating a limiting membrane *in situ*. The mechanisms that regulate lipophagy and how autophagy selectively identifies LDs remain unknown.

Polyubiquitin chains are well-established systems to target proteins and organelles for degradation. The ancient ubiquitous protein (AUP1) interacts with the E2 ubiquitin-conjugating enzyme, Ube2g2, a complex that catalyzes polyubiquitination and is involved in endoplasmic reticulum degradation [115]. AUP1 was found in LDs and it is possible that the AUP1–Ube2g2 complex tags LDs for degradation. Kaushik and Cuervo proposed chaperone-mediated autophagy (CMA). They report that the LD-associated proteins perilipin 2 (PLIN2) and perilipin 3 (PLIN3) that coat LDs are CMA substrates. During starvation, both perilipins increase their expression. Blocking CMA in cell cultures or mouse livers results in decreased lipid oxidation and accumulation of LDs [116].

### Metabolic Diseases Related to Defective Lipophagy

The development of steatosis and fatty liver observed in mice defective for autophagy support the contribution of altered lipophagy to these diseases. In fact, studies have demonstrated that pharmacological upregulation of autophagy reduces hepatotoxicity and steatosis in an alcohol-induced model of fatty liver [117]. Studies in humans with obesity revealed a correlation between autophagic activity and LD size. Protein and mRNA levels of Atg5, LC3-I, and LC3-II were increased in omental fat tissues, and their expression is increased in obese persons, particularly those with intra-abdominal fat accumulation [118]. Lipophagy has also been proposed as a defensive mechanism against arteriosclerosis and accumulation of lipid depots in macrophage foam cells. Foam cells are loaded with cytosolic LDs and represent a hallmark of atherosclerotic lesions. Macrophages located in the subendothelial intima (the innermost layer) accumulate an excess of lipids through scavenger receptors, resulting in foam cell formation. Excess lipid accumulation provokes cytotoxicity, foam cell death, and the release of cytokines, resulting in the migration of new macrophages to the intima region. This is a repetitive process, resulting in the formation of atheromatous plaques of cholesterol, lipids, and cell debris [119]. It has been demonstrated that lipophagy is upregulated in response to lipid loading in macrophages and that the failure to upregulate the autophagic system in mice defective for lipophagy results in inefficient clearance of cholesterol in macrophages [120]. Cancer studies have also focused on lipid metabolism; in fact, a relationship between lipophagy and the tumor suppressor p53 has been revealed. p53 activates lipid hydrolysis and the subsequent oxidation of FAs through the direct control of genes involved in lipophagy and FA β-oxidation [121]. Global genomic profiling revealed that p53 coordinates the expression of several autophagy genes such as ATG7, ATG4, and UVRAG. This study shows that lipid metabolism is regulated by p53 [122]. All these results suggest that the regulation of LDs may be a target for several diseases.

## 6. Conclusions

The increase in the incidence of metabolic diseases associated with impaired lipid metabolism represents a tremendous problem in public health. As we describe, aberrant *de novo* biosynthesis of lipids and also deficiencies or a surplus of specific lipid types derived from the diet can have implications in diseases. Moreover, defective autophagy and lipophagy have been associated with dysfunction of metabolic tissues and may be more complex; these effects need to be elucidated. In this review, we highlight the importance of phospholipids as precursors of almost all cell components involved in the mechanisms ameliorating defects in membrane stability and describe the connection between phospholipids and metabolic diseases. To solve these issues, it is necessary to reinforce the investigation of the crosstalk between biosynthesis and nutrition research. Basic and clinical research is needed to extensively investigate how various diet compositions differentially alter metabolic homeostasis and phospholipid metabolism per se.
